# Common and uncommon vascular injuries and endovascular treatment associated with pelvic blunt trauma: a real-world experience

**DOI:** 10.1007/s11604-022-01355-1

**Published:** 2022-11-09

**Authors:** Ryo Aoki, Kento Nakajima, Yusuke Kobayashi, Yodo Sakai, Hiroyuki Kamide, Toh Yamamoto, Shintaro Furugori, Shungo Sawamura, Miki Terauchi, Kazutoshi Kamiyama, Shin Ikeda, Gengo Tsuji, Shingo Koyama, Jun Yoshigi, Zenjiro Sekikawa, Daisuke Utsunomiya

**Affiliations:** 1 Department of Diagnostic Radiology, Yokohama City University Graduate School of Medicine, Yokohama, Japan; 2 Department of Diagnostic Radiology, Yokohama City University Medical Center, Yokohama, Japan; 3 Department of Diagnostic Radiology, Yokosuka Kyosai Hospital, Yokosuka, Japan; 4 Advanced Critical Care and Emergency Center, Yokohama City University Medical Center, Yokohama, Japan

**Keywords:** Pelvic fracture, Arteriovenous fistula, Arterial occlusion, Vasospasm, Venous bleeding

## Abstract

Pelvic fractures are common in cases of blunt trauma, which is strongly associated with mortality. Transcatheter arterial embolization is a fundamental treatment strategy for fatal arterial injuries caused by blunt pelvic trauma. However, vascular injuries due to blunt pelvic trauma can show various imaging findings other than arterial hemorrhage. We present a pictorial review of common and uncommon vascular injuries, including active arterial bleeding, pseudoaneurysm, arteriovenous fistula, arterial occlusion, vasospasm, and active venous bleeding. Knowledge of these vascular injuries can help clinicians select the appropriate therapeutic strategy and thus save lives.

## Introduction

Pelvic fractures are not rare and account for approximately 3% of skeletal injuries [1]. Pelvic fractures are associated with high mortality risk due to the accompanying vascular injury and hemorrhage [2]. The reported in-hospital mortality rate of patients with unstable pelvic fractures is 8.3% [3].

Transcatheter arterial embolization (TAE) is the fundamental treatment strategy for patients with fatal arterial injuries caused by blunt pelvic trauma [4, 5]. However, these patients present with various findings other than arterial hemorrhage, which may hinder interventional radiologists from determining an appropriate treatment strategy. We present a pictorial review of real-world experiences of common and uncommon vascular injuries, including active arterial bleeding, pseudoaneurysm, arteriovenous fistula (AVF), arterial occlusion, vasospasm, and active venous bleeding.

## Types of vascular injuries

### Active arterial bleeding

TAE is the fundamental treatment strategy for active arterial bleeding due to pelvic fractures [4]. Embolization of the main trunk or branches of the internal iliac artery is the basic endovascular treatment. In cases of shock or multiple traumas, non-selective embolization of the internal iliac artery should be performed [6]. Selective embolization of the internal iliac artery branch can be considered when the patient’s hemodynamics are stable, and there is enough time to perform the procedure. Furthermore, it is necessary to look for bleeding from sources other than the internal iliac artery. Bleeding from the branches of the external iliac artery reportedly occurs in 17% of patients with pelvic fractures [7]. Angiography of the external iliac, median sacral, lumbar, and femoral arteries should be performed.

Embolic material is chosen according to the bleeding site and the presence or absence of coagulation abnormalities. Gelatin sponge particles are often used as embolic materials to avoid permanent arterial occlusion and ischemia. Coils may be used when the bleeding vessels are focal and the catheter can reach the vicinity of the bleeding sites. In coagulopathic patients, liquid embolization with *n*-butyl cyanoacrylate (NBCA) is useful for both selective and non-selective embolization; however, an experienced operator is desirable to prevent NBCA-related complications, such as reflux and non-target embolization [8].

**Fig. 1:**
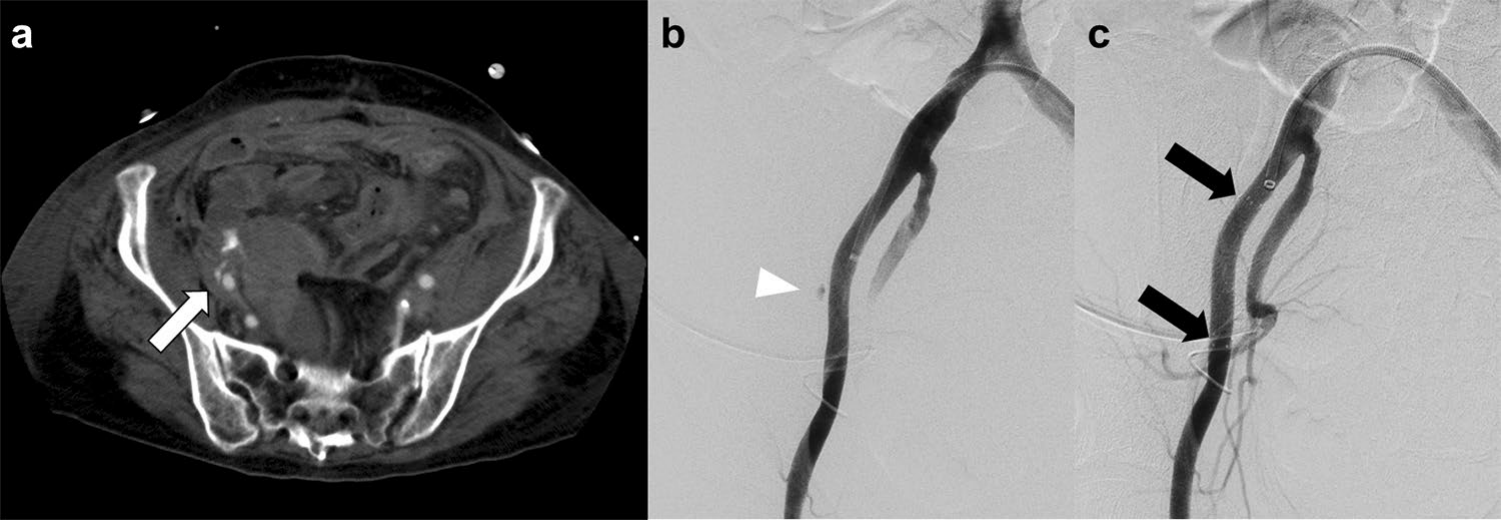
Endovascular treatment with stent graft placement for active bleeding from the external iliac artery. **a** CT scan shows active bleeding from the right external iliac artery (white arrow). **b** Digital subtraction angiography of the right external iliac artery shows active bleeding (arrowhead). **c** Digital subtraction angiography of the right external iliac artery obtained after angioplasty with stent graft placement (VIABAHN®, W. L. Gore & Associates, Flagstaff, Ariz) shows cessation of active bleeding. Black arrows indicate the markers of the proximal and distal edges of the stent graft. *CT* computed tomography

**Fig. 2:**
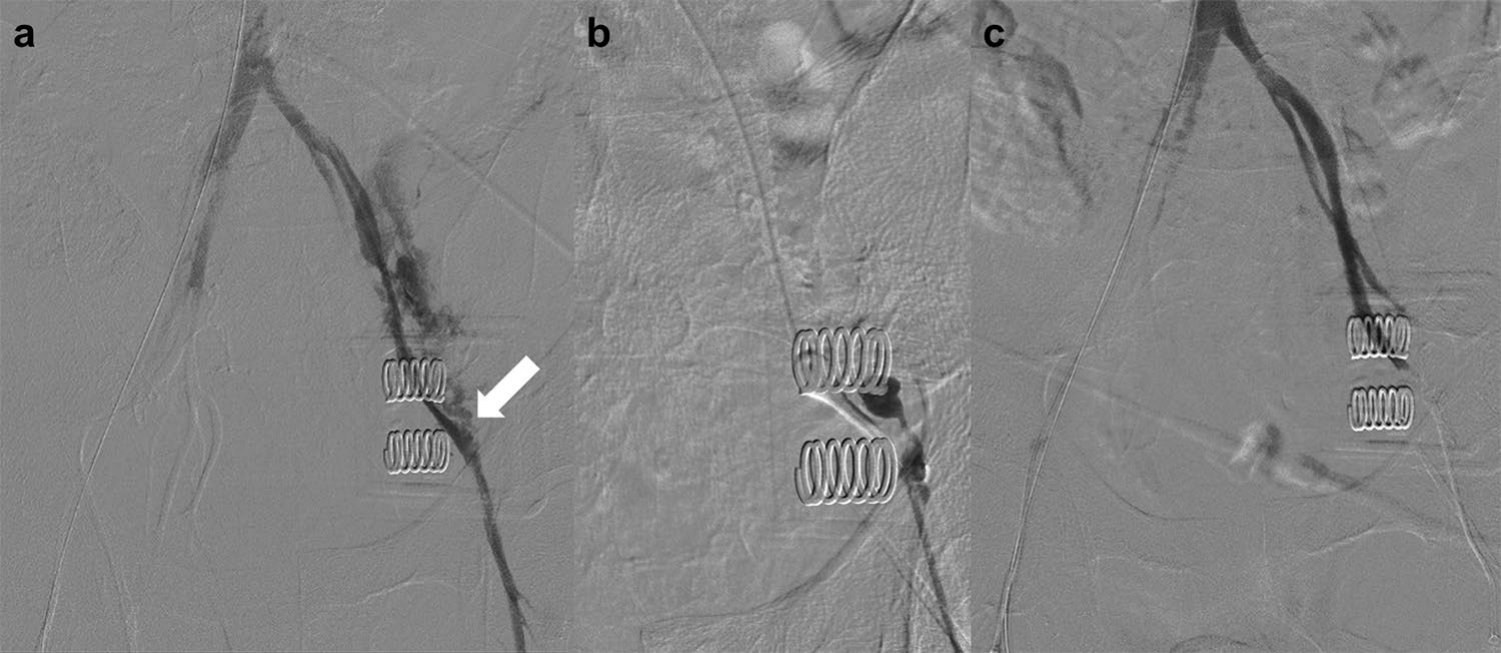
Active bleeding from the external iliac artery due to a crush injury in a 54 year-old man. This patient experienced cardiopulmonary arrest during the procedure and required prompt hemostasis because circulation could not be restored with REBOA alone. **a** Digital subtraction angiography of the left external iliac artery shows active bleeding (arrow). **b** The bleeding point has been embolized with NBCA. NBCA was mixed with iodized oil in a ratio of 1:1. **c** Digital subtraction angiography of the left external iliac artery obtained after embolization shows cessation of active bleeding and occlusion of the external iliac artery. However, the patient died after the procedure without recovery of circulation. *REBOA* resuscitative endovascular balloon occlusion of the aorta; *NBCA* *n*-butyl cyanoacrylate

Considering the risk of lower extremity ischemia after TAE of the main trunk of the common iliac and external iliac arteries, stent graft treatment can be used to stop the bleeding (Fig. 1) [8]. Antiplatelet therapy should be initiated in patients receiving stent graft treatment to prevent post-procedure graft occlusion. However, there is no consensus on the timing of the initiation and termination of the antiplatelet medication. In clinical emergencies, where prompt treatment is needed to save the patient’s life, TAE of the common and external iliac arteries may be considered despite the high risk of lower extremity ischemia (Fig. 2).

**Fig. 3:**
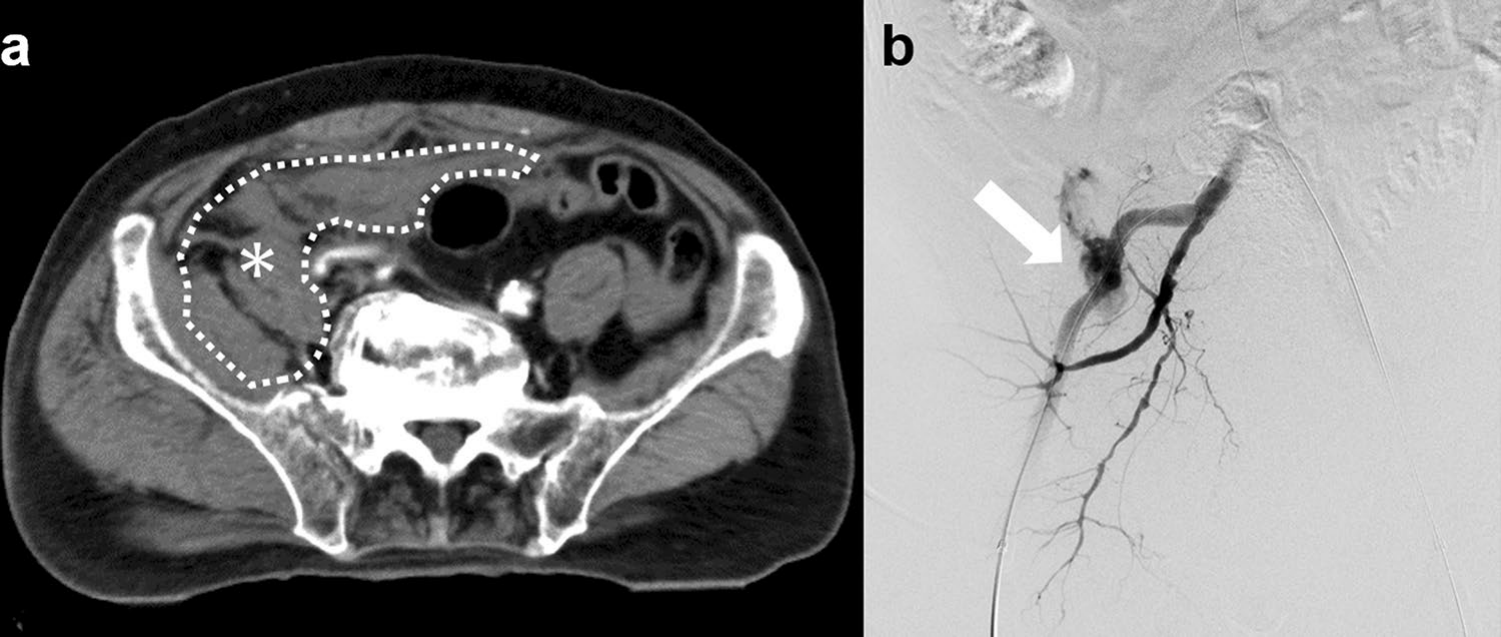
Pelvic contrast-enhanced CT images obtained during complete REBOA. **a** Delayed-phase (second phase) contrast-enhanced CT image shows a massive hematoma (asterisk) without extravasation of the contrast material. **b** Digital subtraction angiography of the right internal iliac artery obtained during REBOA balloon deflation shows massive extravasation from the iliolumbar artery, which represents “hidden” bleeding on contrast-enhanced CT during complete REBOA (arrow). We embolized the iliolumbar artery with NBCA. *CT* computed tomography; *REBOA* resuscitative endovascular balloon occlusion of the aorta; *NBCA* *n*-butyl cyanoacrylate

Resuscitative endovascular balloon occlusion of the aorta (REBOA) is an important method for treating severe hemorrhagic shock due to pelvic trauma. In this procedure, occlusion of the aorta with a balloon catheter is conducted to increase proximal arterial pressure and maintain organ blood flow while controlling downstream bleeding [9]. However, REBOA can lead only to temporary hemostasis and is associated with a risk of organ ischemia. Definite hemostasis should be achieved with endovascular or surgical intervention, and the balloon should be deflated promptly to ensure sufficient blood supply to the organs. Furthermore, it should be noted that contrast-enhanced computed tomography (CT) may not demonstrate the extravasation of contrast medium as a result of temporary hemostasis of active bleeding by REBOA (Fig. 3) [10, 11]. In particular, the flow of the contrast medium into the site of active bleeding may be slowed due to the balloon occlusion, resulting in “hidden” bleeding (Fig. 3a). The optimal timing of delayed phase CT is difficult to determine, and the CT protocol for REBOA remains unestablished. We posit that multiple phase CT, including super-delayed phase imaging, may be useful. Further studies should be imperative.

### Pseudoaneurysm

The formation of pseudoaneurysms in patients with blunt pelvic trauma, although rare, may occur immediately after the injury or later in the clinical course [12]. Pseudoaneurysms are made of a single layer of fibrous tissue and are often surrounded by a hematoma. [13]. They can rupture and should be treated as soon as they are identified [14]. On CT, a pseudoaneurysm appears as a sac filled with contrast material in the arterial phase, and it is stable in size and shape in the delayed phase, disappearing gradually [15].

**Fig. 4:**
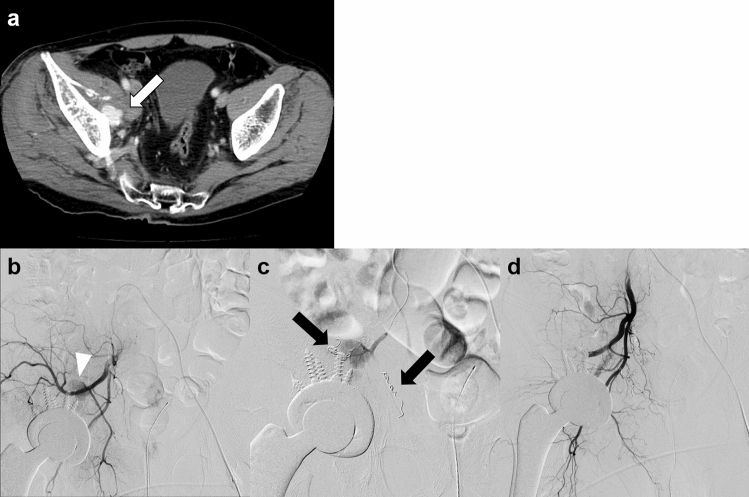
Pseudoaneurysm due to pelvic trauma in a 67 year-old man. **a** CT scan obtained 2 weeks after trauma shows pseudoaneurysm (arrow). **b** Digital subtraction angiography of the superior gluteal artery shows pseudoaneurysm (arrowhead). **c** Embolization of the obturator artery and distal portion of the superior gluteal artery has been performed with microcoils (black arrows) to avoid migration of NBCA to the peripheral arteries. **d** Selective embolization of the pseudoaneurysm with NBCA has been performed; digital subtraction angiography of the internal iliac artery shows the disappearance of the pseudoaneurysm. *CT* computed tomography; *NBCA* *n*-butyl cyanoacrylate

Endovascular treatment of traumatic pseudoaneurysm is usually effective (Fig. 4) [16]. The sandwich method is used for TAE of pseudoaneurysms, i.e., embolization of the afferent and efferent vessels to cut off the blood supply to the lesion [17]. However, in this method, the parent blood vessel is occluded. Sac packing with detachable microcoils is another option for pseudoaneurysm treatment, and it preserves the parent arterial patency; however, this procedure is associated with the risk of secondary rupture [18]. In pseudoaneurysms without multiple collateral vessels, stent graft treatment can be considered to preserve the parent vessel [19]. In addition, stent graft treatment may also be effective in cases of failed sac packing.

### AVF

AVF is an abnormal anastomosis between arteries and veins and does not involve capillaries. Traumatic AVF can develop if arterial bleeding influxes into an adjacent injured vein [15]. On radiological imaging, early filling of a pelvic vein with contrast material may be the only finding in patients with traumatic AVF [20]. Radiologists should recognize the angiographic pseudovein sign that typically indicates gastrointestinal bleeding (extravasation of contrast agent), which may appear curvilinear, mimicking the appearance of veins [21]. It is important to differentiate the pseudovein sign from AVF for appropriate treatment selection. However, the clinical diagnosis of traumatic AVF is often delayed, and the rate of diagnosis within 1 week of injury is reported as only 20% [22]. The cause of the delayed AVF development is unclear. However, delayed AVF may occur when a potentially causative arterial bleeding is undetected and untreated, or when the initial endovascular treatment is inadequately embolized. Therefore, knowledge of traumatic AVF imaging features is essential for radiologists.

**Fig. 5:**
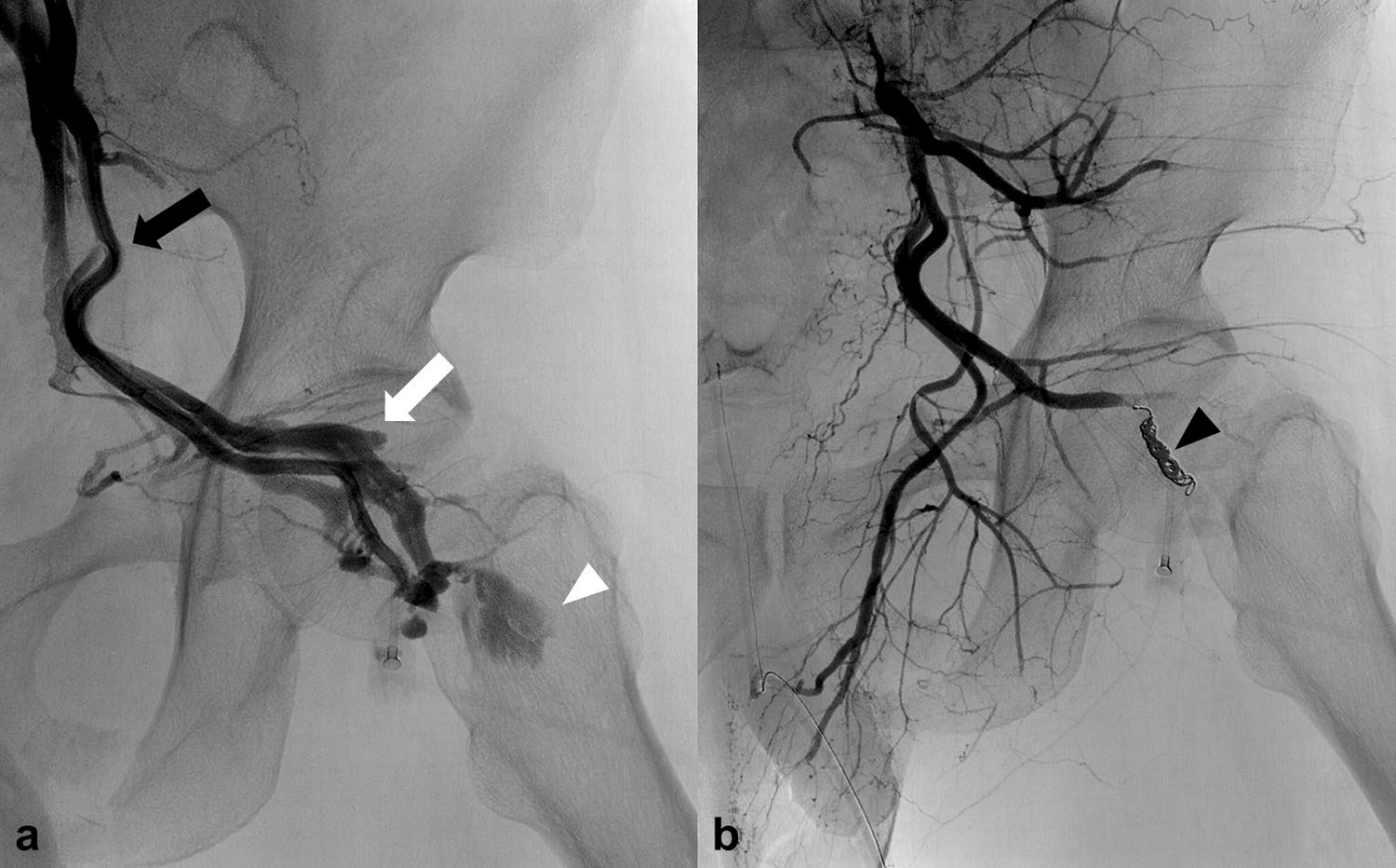
AVF due to a fall in a 43 year-old man. **a** Selective angiography of the inferior gluteal artery shows an AVF with active bleeding (white arrowhead). The black and white arrows indicate the inferior gluteal artery and inferior gluteal vein, respectively. **b** The main feeding artery of the AVF has been embolized with microcoils (black arrowhead). In addition, small branch arteries have also been embolized by injecting gelatin particles into the origin of the inferior gluteal artery. Subsequently, AVF and active bleeding disappeared. *AVF* arteriovenous fistula

**Fig. 6:**
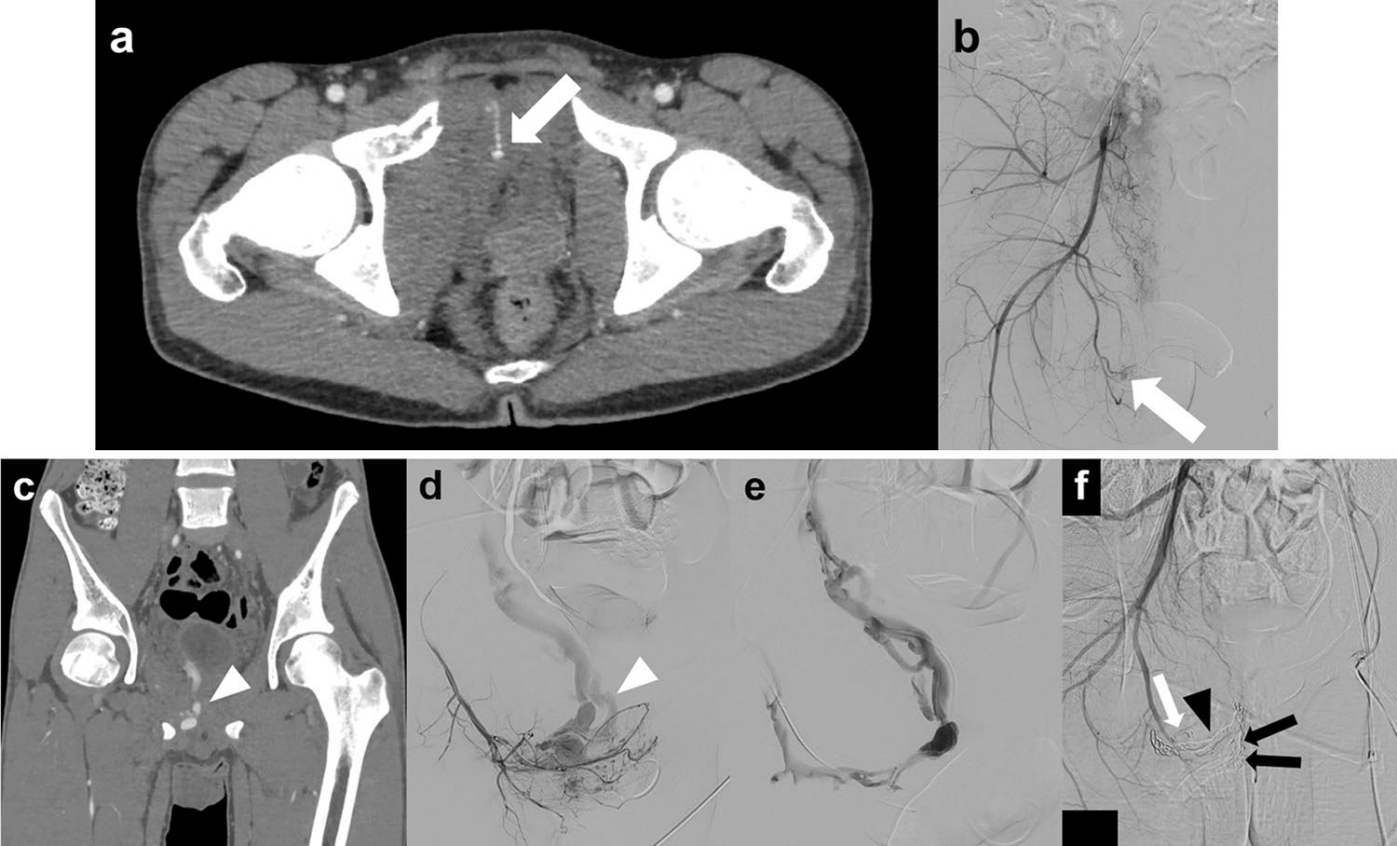
Delayed AVF due to a traffic accident in a 17 year-old boy. **a**, **b** CT scan and digital subtraction angiography of the right internal iliac artery obtained immediately after admission show active bleeding (arrows). Non-selective embolization of the right internal iliac artery with gelatin particles has been performed. **c**, **d** Coronal view CT scan and digital subtraction angiography of the internal pudendal artery obtained 3 weeks after injury show early filling of a pelvic vein (arrowhead). The AVF has multiple feeding arteries. **e** A transvenous approach has been used to embolize the drainer vein. **f** The main drainer vein has been embolized with microcoils (arrowhead), and the feeding arteries have been embolized with NBCA (black arrows), gelatin particles, and microcoils (white arrow). Digital subtraction angiography of the right internal iliac artery shows disappearance of the AVF. *AVF* arteriovenous malformation; *CT* computed tomography

Because traumatic AVF can cause heart failure after highly variable latency periods [23], AVFs should be treated regardless of the presence of active bleeding. TAE may be a safe and efficient treatment method for traumatic AVF (Fig. 5) [24–26]. Embolic materials should be selected carefully to prevent migration to the venous flow. When the AVF lesion has multiple feeding arteries, concomitant transarterial and transvenous embolization may be an effective option (Fig. 6) [27].

### Vasospasm

**Fig. 7:**
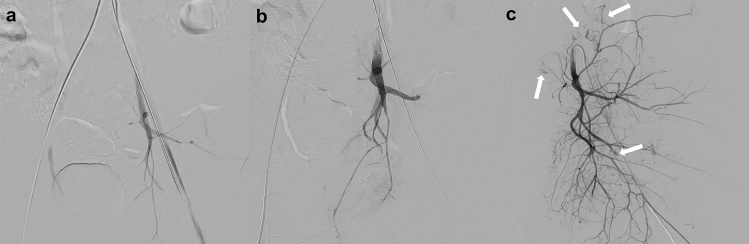
Arterial vasospasm after a traffic accident in a 29 year-old man with pelvic fractures and thoracic injury. **a** Digital subtraction angiography of the left common iliac artery shows arterial vasospasm. Peripheral branches of the internal iliac artery are poorly depicted, and active bleeding cannot be detected. **b** Digital subtraction angiography obtained after non-selective embolization of the internal iliac artery with gelatin particles shows no active bleeding. Thoracic surgical procedures have been performed to ensure hemostasis after the first embolization session. **c** The second session of endovascular treatment has been performed because of unstable hemodynamics after thoracic surgery. Digital subtraction angiography of the left internal iliac artery shows scattered active bleeding due to the release of vasospasm

The middle layer of blood vessels is made up of smooth muscles, and smooth muscle cells may contract and become spasmodic following external compression, stretching, or endothelial injury due to trauma [28]. Active bleeding is not usually detected on the first angiogram due to vasospasm, but it can be detected through repeated angiography [29, 30]. Recurrent bleeding from a previously embolized artery can also be explained by vasospasm. With improved vessel diameter after resuscitation, the embolic material might not occlude the bleeding vessel (Fig. 7) [30, 31]. Therefore, patients with unstable blood pressure should undergo repeated angiography regardless of whether embolization was performed. To prevent rebleeding in coagulopathic patients with vasospasm, we recommend embolization with NBCA, which adheres to the vessels and is unlikely to migrate.

### Arterial occlusion

**Fig. 8:**
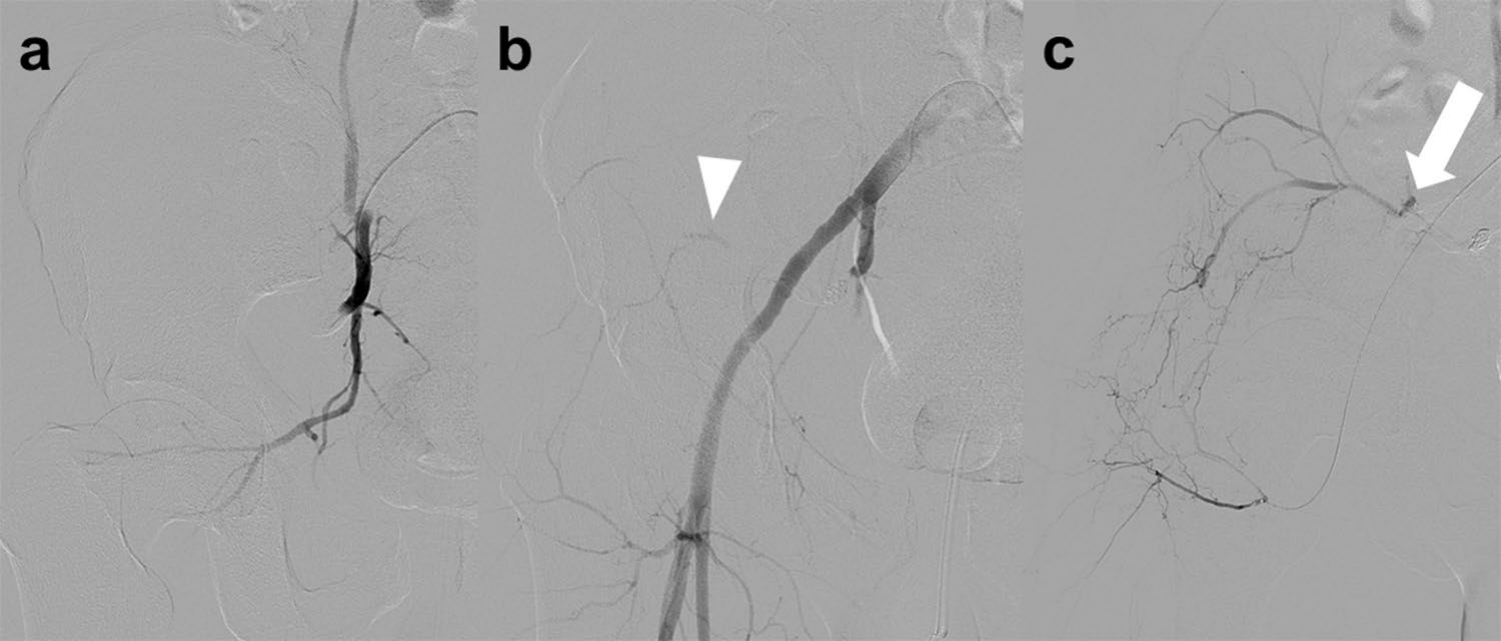
Arterial obstruction due to a crush injury in a 50 year-old man. **a** Digital subtraction angiography of the right internal iliac artery shows complete obstruction of the superior gluteal artery. **b** Embolization with microcoils of the superior gluteal artery and non-selective embolization with gelatin particles of the internal iliac artery have been performed. Digital subtraction angiography of the right common iliac artery obtained after embolization of the right internal iliac artery shows blood flow from a branch of the external iliac artery to the distal portion of the occluded superior gluteal artery (arrowhead). **c** Digital subtraction angiography of the lateral femoral circumflex artery shows collateral circulation to the distal portion of the superior gluteal artery with extravasation of contrast material (arrow). Embolization with gelatin particles led to successful hemostasis

Arterial occlusion is caused by transmural injury or dissection with a thrombus [15]. Active bleeding is a serious concern in patients with arterial occlusion, even if extravasation of contrast material is not detected on CT [20]. In addition, traumatic arterial occlusion has the potential pathogenesis of delayed bleeding due to the mobilization of the thrombus [32]. Therefore, TAE of the occluded arteries in the branches of the internal iliac artery may be appropriate to prevent rebleeding. Furthermore, angiography of the collateral blood vessels should be performed to check for active bleeding distal to the occluded vessel (Fig. 8). In contrast, stent graft treatment for traumatic vessel occlusion is controversial, and further studies are warranted.

Traumatic occlusion of the main trunk of the common/external iliac artery can cause critical lower limb ischemia, making recanalization crucial [33]. Vascular surgery is a treatment option for traumatic occlusion of the main trunk of the common/external iliac artery. Furthermore, endovascular treatment with covered stents, aspiration thrombectomy, and balloon dilation can also be performed [34, 35]. In addition, using a cutting balloon may be helpful when intimal thickening is present at the traumatic occluded site [35].

### Active venous bleeding

Bleeding due to pelvic fractures occurs more frequently from veins (80%) than from arteries (20%); the main venous sources of bleeding are the presacral plexus and prevesical veins [36]. Peritoneal pelvic packing and mechanical pelvic ring fixation can be performed to treat venous bleeding [37]. Endovascular treatment may be optimal for active bleeding from the main trunk of the iliac vein.

**Fig. 9:**
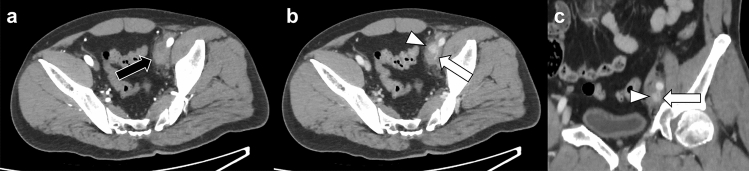
Venous injury due to a traffic accident in a 50 year-old man. **a** Arterial phase pelvic CT scan shows no extravasation of contrast material within the hematoma (black arrow). **b**, **c** Venous phase CT scan (**b**: axial view, **c**: coronal view) shows extravasation of contrast material (arrowheads) with a narrowed external iliac vein (arrows). CT, computed tomography

Active venous bleeding can be visualized using CT as the extravasation of contrast material in venous phase images without extravasation in arterial phase images (Fig. 9) [20]. Iliac vein injury causes massive bleeding and hemorrhagic shock, and venography is useful in detecting active bleeding from the iliac vein in some patients with blunt pelvic trauma [38]. Surgical or endovascular therapies can be used for treating iliac vein injuries.

Transcatheter venous embolization with NBCA can potentially control critical bleeding from the iliac vein. However, it may cause some complications, such as leg swelling, deep vein thrombosis, and pulmonary embolism by obstruction of venous flow [39]. Endovascular treatment with covered stents is considered a favorable treatment option [40, 41], if an appropriately sized stent is readily available. When a covered stent is not immediately available, uncovered stent placement is an alternative option for endovascular treatment for critical iliac vein injury [38, 42, 43]. Stenting of venous injuries covers the injured area and promotes hemostasis by reducing venous pressure, which is elevated because of the surrounding hematoma [43].

When surgical treatment is chosen for a venous injury, balloon catheters can reduce bleeding and help obtain the necessary visualization of the operative field by occluding the proximal and distal portions of the injury [44].

## Conclusion

Endovascular treatment is effective for vascular injuries due to blunt pelvic trauma. Knowledge of the various forms and imaging findings of vascular injuries is essential for the astute planning of treatment strategies and life-saving maneuvers.
